# Reinventing “N” in the A/T/N framework: The case for digital

**DOI:** 10.1016/j.tjpad.2025.100395

**Published:** 2025-12-01

**Authors:** Rhoda Au, Zachary Popp, Spencer Low, Nicholas J. Ashton, Henrik Zetterberg

**Affiliations:** a Department of Anatomy & Neurobiology, Boston University Chobanian & Avedisian School of Medicine, Boston, MA, USA 02118; b Boston University Alzheimer's Disease Research Center, Boston University Chobanian & Avedisian School of Medicine, Boston, MA, USA 02118; c Departments of Neurology, Medicine, and Framingham Heart Study, Boston University Chobanian & Avedisian School of Medicine School of Medicine, Boston, MA, USA 02118; d Department of Epidemiology, Boston University School of Public Health, Boston, MA, USA 02118; e Department of Psychiatry and Neurochemistry, Institute of Neuroscience & Physiology, the Sahlgrenska Academy at the University of Gothenburg, 405 30 Mölndal, Sweden; f Banner Alzheimer's Institute and University of Arizona, Phoenix, AZ, USA; g Banner Sun Health Research Institute, Sun City, AZ 85351, USA; h NIHR Biomedical Research Centre for Mental Health and Biomedical Research Unit for Dementia at South London and Maudsley NHS Foundation London SE5 8AZ, UK; i Centre for Age-Related Medicine, Stavanger University Hospital, 4068 Stavanger, Norway; j Clinical Neurochemistry Laboratory, Sahlgrenska University Hospital, 413 45 Mölndal, Sweden; k Department of Neurodegenerative Disease, UCL Institute of Neurology, Queen Square, London WC1N 3BG, UK; l UK Dementia Research Institute at UCL, London WC1N 3BG, UK; n Hong Kong Center for Neurodegenerative Diseases, Clear Water Bay, Hong Kong Science Park, Shatin, N.T., Hong Kong, China; m Wisconsin Alzheimer’s Disease Research Center, University of Wisconsin School of Medicine and Public Health, University of Wisconsin-Madison, Madison, WI 53792, USA

**Keywords:** A/T/N framework, Technology, Digital “N”, Alzheimer’s disease, Biomarkers, Clinical meaningfulness

## Abstract

Breakthroughs in biomarkers for amyloid (A), tau (T), and neurodegeneration (N) have advanced the prospects of accurate Alzheimer’s disease (AD) diagnosis. However, presence of pathology does not always translate into clinical expression and there are still clear knowledge gaps as to whether someone with AD biological indicators will lead to clinically apparent disease necessary to warrant drug treatments that carry toxicity risk. Reliance on decades-old assessment tools inhibits detection and monitoring at preclinical and early disease stages when new treatments could prove most effective. Evidence has accumulated that digital measures provide accurate detection of disease at early stages. We call for a re-evaluation of the A/T/N diagnostic framework, with digital evaluation measures complementing non-AD specific neurodegeneration markers, and even potentially replacing those non-specific to AD, to provide a clinically relevant feature critical to clinical trial advances and treatment decisions. Achieving this will only be possible if further research into novel digital evaluation tools is pursued with the same support and consideration as amyloid and tau.

## Current state of the A/T/N framework

Accurate diagnosis at the primary care level has been one of the holy grails of Alzheimer’s disease (AD) research. The United States’ (US) Food and Drug Administration (FDA) 501(k) clearance of cerebrospinal fluid (CSF) biomarkers of amyloid (A) and tau (T) [1], the hallmark pathologies of AD, brought that goal within reach, but still within the remit of specialist care. Recent FDA clearance of the Lumipulse G pTau217/amyloid 1-42 ratio as a blood-based biomarker for AD, with more plasma AD platforms in the FDA approval pipeline [2], marks the remaining step to widespread clinical utility. AD blood-biomarkers will enable primary care health providers to identify patients at risk for AD [3] Accumulating data show CSF equivalence of many of the simplified blood tests in clinically relevant settings [4], and secondary analyses of key trials further demonstrating the promise of plasma biomarkers as pharmacodynamic markers of AD treatments, such as p-tau217 in the TRAILBLAZER study [5]. Simplified sampling and pre-analytical sample handling through dried plasma spot analysis create opportunities to deploy at scale [6]. Neurodegeneration (N) rounds out the A/T/N framework for diagnosis of AD distinct from other dementia subtypes [7], and can be indicated through magnetic resonance imaging (MRI) atrophy, fluorodeoxyglucose positron emission tomography (FDG-PET) metabolism, or several other markers, but obtaining these measures require access to facilities and are costly. Plasma neurofilament light (NfL) has been linked to AD neurodegeneration [8] and its FDA Breakthrough Device Designation status has led to NfL being a marker of N, that can much more easily be measured at scale. It should be noted, however, that NfL is also ubiquitously increased in most neurodegenerative disorders [9] and a primary outcome in multiple sclerosis (MS) and amyotrophic lateral sclerosis (ALS) drug efforts. Also of note is that the FDA- approved plasma biomarkers for specificity of AD do not include any general marker of neurodegeneration. Therefore, while NfL as well as glial fibrillary acidic protein (GFAP) have been promoted as part of the AD plasma 94 biomarker diagnostic panel, they are not critical for diagnosis of AD. Worldwide, given that people over the age of 65 now outnumber those under the age of 5, projected cases of AD are expected to greatly expand by 2050. Thus, the availability of AD plasma biomarkers makes possible an easily accessible and scalable AD diagnosis tool at greatly reduced costs, there democratizing AD research, and treatment care that currently suffers from bias towards the high income, highly educated and well-insured [10].

Spurred by the National Alzheimer’s Project Act (NAPA) [11] established in 2012 under the Obama Administration, the secondary goal of finding effective treatments by 2025 is on track. Despite the many debated limitations, aducanumab was the first approved drug for treatment of AD since NAPA was constituted, and in the context of a precision medicine approach, appears to modify disease symptoms for a small subset of patients with early AD and PET-confirmed high amyloid [12]. Moreover, lecanemab received FDA accelerated approval in 2023 based on promising findings [13], and donanemab soon followed with its own FDA approval [14]. Readouts from other Phase 3 clinical trials are expected over the next few years setting the stage for other FDA approved AD treatment options in the near future. Further, the spate of Phase 1 and Phase 2 clinical trials has grown substantially, fueled in part by the additional investment in AD research by the National Institute on Aging. In combination, the progress that has been made in AD over the past decade is impressive. These successes demonstrated critical gaps that need to be addressed to maximize the benefit of recent scientific headways.

## Limitations to the A/T/N framework

Autopsy-confirmed A/T/N is not always accompanied by concomitant clinical expression [15]. While it is widely presumed that antemortem A/T/N biomarker positivity is an indicator for preclinical or MCI due to AD detection, it is less clear whether presence of AD pathology will lead to clinically expressed disease. Much of the data available is on non-representative populations, whether it be from post-mortem study samples or from antemortem CSF, /PET/MRI studies. Given this known longstanding recruitment bias in AD research, stemming from a subset having the means or willingness to participate in studies that are based at a high resourced research environment, what remains unknown is how prevalent is AD pathology in the absence of clinical symptom in the general population. This gap in knowledge is important because recently FDA approved AD treatments carry significant toxicity risk that includes premature death. In general, risk of significant side effects is typical for any drug treatment. Thus, regardless of the number of AD drug treatments that make it to market, a perpetual question for any healthcare provider is under what circumstances would AD treatment be warranted, particularly if there is an absence of clinically apparent symptoms. Plasma AD biomarkers, which are highly sensitive to AD, still suffer from variability in specificity. If research documents even greater prevalence of AD biomarkers that is clinically silent in the general population than is currently known, the bar may likely be raised for evidence for clinically symptomatic indicators of disease [16]. Further, use cases for treatment and clinical trial eligibility are complicated by the heterogeneity identified in biomarker positivity and/or clinical presentation among patients with different demographic characteristics, including sex, education or racial and ethnic backgrounds [17]. Thus, despite the premise that early detection enabled by AD plasma biomarkers could potentially allow intervention at a time when treatment would be most effective, an ethical question is raised as to the appropriateness in trial enrollment, and eventually treatment, for those who are A/T/N biomarker-positive in the absence of any clinical indicators. We contend that detection of biomarkers must be accompanied by clinical symptoms to avoid treating individuals who may be misdiagnosed. For drug treatments that carry some level of toxic risk, reliance on biomarker positivity alone could be tantamount to giving drug treatment to any person who has any other risk factor in which AD risk is increased but does not result in AD that meets threshold for clinically diagnosed disease. For example, those who are Apolipoprotein e4 allele (APOE4) heterozygote or homozygote positive, their increased risk for AD is 2-3 or up to 10-15 fold higher that those who are *APOE4*-negative but clinical AD diagnosis is not 100 % inevitable. Similarly, those with high cardiovascular risk can be as high as triple the risk for AD compared to those with low cardiovascular risk but are not certain to developed clinically diagnosed AD.

This ethical issue is unlikely to be realized in the near future given the lack of available treatments at preclinical disease stage also known a mild cognitive impairment (MCI) due to AD. Recent advances, however, demonstrate how quickly new treatments may become available to patients. Solutions for detection at the preclinical disease stages would provide a new population for clinical trial enrollment with early intervention. An additional benefit is the potential to identify individuals to prioritize for non-pharmacological interventions. The current A/T/N framework does not have the specificity to detect the nuance of an inherently insidious clinical onset process that spans from presumably asymptomatic (using current clinical diagnostic tools) to the clinically symptomatic.

While A/T are largely accepted as specific to AD diagnosis, regardless of the why and how [[18], [19], [20], [21]], the same cannot be said about N. NfL is just one of several different plasma biomarkers of neurodegeneration [22,23], but given its widespread use, it has emerged as a well-accepted measure of neurodegeneration. NfL has been documented as an indicator of neuronal injury that is not only evident in multiple sclerosis and AD but also other neurodegenerative disorders, as well as in acute conditions, including cardiac arrest [24], stroke, brain trauma and encephalitis [25]. Further, blood NfL is also affected for those with chronic kidney disease and by BMI, [26,27] complicating the use of this more accessible marker in clinical practice. Given the non-specificity of any “N” biomarker, including NfL and the previously described, concerns around the utility of A/T/N for determining AD specificity, clinical trial eligibility and appropriate treatment options encompassing all individuals, an opportunity emerges to step out of conventional A/T/N biomarker lanes.

## Importance of clinically meaningful endpoints

Clinical meaningfulness is generally applied as an outcome measure of import in clinical trials studies that seek FDA approval. For AD-related clinical trials, the administration of neuropsychological tests, such as the Mini-Mental State Examination (MMSE) or Alzheimer’s Disease Assessment Scale-Cognitive Subscale (ADAS-Cog), are routinely used to generate metrics of cognition, one of the key performance indicators of clinical trial intervention effectiveness. Until the aducanumab approval, a component of the FDA AD drug treatment approval hinged on demonstrating either improvement or attenuated decline/no further decline in cognitive function. This determination was often made through a composite or domain specific neuropsychological test score (e.g., memory) as a proxy for clinical meaningfulness. The Centers for Medicare and Medicaid Services (CMS) decision to restrict coverage of aducanumab, and its subsequent withdrawal from the market, was due to the lack of sufficient evidence related to neuropsychological measures of cognition despite its measured statistical significance (22 % reduction in decline of clinical dementia rating in high-dose arm vs placebo) [28,29]. At the heart of the controversy related to FDA approvals for aducanumab is the recognition that clinical manifestation of AD is crucial and that cognition is a critical feature of clinical manifestation. The increased receptivity to lecanemab and donanemab both within and outside of the U.S., stems, in part, from the slowed progression of cognitive decline by 27 % and 33 %, respectively, within those in the treatment group compared to the placebo group [30] although to what extent this attenuation will persist beyond the original trial study period is still being determined.

## Perpetuating assessment bias in clinically meaningful endpoints

The significance of cognition in any AD related clinical trial makes it all the more surprising how little has been done to advance the field of cognitive assessment. Clinical trials have relied on one or multiple tests that were developed decades ago (e.g., ADAS-cog, MMSE, Montreal Cognitive Assessment [MoCA], and CERAD [Consortium to Establish a Registry for Alzheimer’s Disease]) [31], despite recognition of the constraints of these instruments [32]. Limitations include ceiling effects, invariances, reliability, and appropriateness for racially, ethnically, educationally or language diverse populations [33]. Advances in these instruments since their development are limited. Several large-scale trials have aimed to address the issues with traditional tools by adding supplementary tests [34] or exploring alternative scoring methods [35]. Alternatively, clinical trials have explored constructing composite batteries from subsections of the ADAS-cog and other instruments [36].

These efforts do not address the root cause of the shortcomings in cognitive assessment. ADAS-cog was developed in 1984 [37] the MMSE was developed in 1975 [38] and the CERAD in 1989 [39]. The inability to rethink effectively measuring cognition hinders the ability to detect efficacy in clinical trials and impacts who enrolls based on testing-based inclusion and exclusion criteria. High screen-fail rates have been addressed previously by swapping one traditional tool for another [40]. The minor iterations of decades-old instruments have resulted in heterogeneity of tests, with 31 known versions of the ADAS-cog instrument as of 2018 [32]. Additions of subscales further exacerbate the existing concerns of traditional assessment methods. The widespread practice of simply translating and making minor modifications to the assessments for use outside of North America and Western Europe has perpetuated the educational, cultural, and linguistic bias inherent in these tests. As efforts to develop effective translations have grown, there remains inconsistency in the standards for item-level modifications, and the lack of normative data continues to lead to cultural biases [41].

## First call to action: invest in digital to develop more sensitive and less biased clinical measures

Measures currently described as “digital biomarkers” would be more accurately described as “digital phenotyping”. Digital phenotyping describes the moment-by-moment quantification of behavior using embedded digital sensors, such as smartphones [42]. Many digital phenotyping approaches for cognitive assessments rely on active engagement assessments that require a person to respond to questions similar to standardized neuropsychological tests. For example the Framingham Heart Study deployed a smartphone application that collects voice and response to test stimuli or finger responses to screen-based tests. The Intuition Study, that was more nationally representative, including more geographically balanced, used the Cambridge Cognition smartphone application (CamCog) as a digital cognitive assessment. These and other studies are able to collect additional digital phenotyping measures through embedded sensors, supplementing more traditional neuropsychological test measures. This approach, however, does not provide more highly sensitive detection of subtle clinical changes that will more accurately differentiate those that might be AD biomarker positive and largely asymptomatic versus those who are AD biomarker position but showing early signs of MCI due to AD stage. This distinction between AD biomarker positive but asymptomatic versus MCI (or preclinical/prodromal AD) is important. The concept of “asymptomatic” itself is highly dependent on the sensitivity of the tools being used to measure symptom. Cognitive assessments acquired using digital tools show promise for detecting differences in cognitive performance to a greater extent than standardized test scores including through multimodal measurement, which brings together different digital data modalities to measure a series of interrelated functional and behavioral measures [43]. As digital phenotyping becomes more integrated into AD research, there is a likelihood that a recognized separation between “asymptomatic” versus “preclinical” will begin to emerge, just as the distinction between MCI and mild AD is now accepted. A growing number of studies are deploying digital tools to do more than derived measures that mimic well-established measures that detect cognitive AD-related manifestations. The undisputed pioneer in this realm has been the work of Kaye and colleagues, who were the first to test embedding sensors as an alternative to studying longitudinal trajectories of behavior change in the home [44]. They found that frequent sensor-based monitoring allowed for more accurate detection of the transition from normal cognition to MCI [44]. In addition, Kaye et al. monitored elderly participants answering online surveys and demonstrated slowed survey completion time preceded the onset of MCI by over a year [45]. In another study of 27 participants who were not demented, less daily computer use was associated with smaller hippocampal volume, a well-established neuroimaging biomarker of neurodegeneration associated with increased AD risk [46]. In the first study to harness the natural behavior of voice, Kaye et al. [47], utilized remote video telecommunication software to record average talk time per day to determine whether speech detection algorithms could determine normal cognitive aging to MCI transitions. They reported that MCI subjects spoke more words during conversations and exhibited longer daily talking times than normal subjects. They concluded that MCI subjects exhibit subtle language processing deficits that are sensitive to transitions to MCI. Home monitoring with infrared sensors has also been used to classify sleep, activity, gait, and behavioral changes relevant to neurodegeneration and traumatic brain injury. Through capturing novel metrics of behavior, use of mobile technologies affords the opportunity for longitudinal remote data collection with evaluation occurring more frequently and with greater granularity. Importantly, these measures can offset some of the active engagement assessment bias because they are collected from any person’s use of these technologies’ independent of their age, education, language or culture.

The unique importance of the early studies done by Kaye et al. is they repurposed technology to measure AD-related cognitive behaviors. while avoiding the inherent bias of traditional assessment tools through a focus on unstructured data streams like voice, and longitudinal behavioral change in a real-world setting. The evolution from computers to smartphones has lowered the barriers to assessment for those who live far from healthcare facilities/providers with the training or professional expertise to administer them. A notable effort by the Real-World Implementation, Deployment, and Validation of Early Detection Tools and Lifestyle Enhancement (AD-Riddle) project has reviewed and tested digital platform for multi-national deployment. While there are numerous similar efforts underway that are addressing the cross-cultural considerations, many assessments do not adequately address the importance of language on performance. There are an estimated 7159 languages in the world today, and no single cognitive assessment tool that is available in all these languages. With the anticipated increase in AD incidence and prevalence across the world, the lack of an assessment tool that could be applicable to anyone, anywhere will further exacerbate the significant global health inequities in clinical practice and research [48]. Building on the monitoring of health during daily behavior first demonstrated by Kaye et al. is expected to reduce biases in that evaluation of cognition and function need not rely on specific stimuli requiring specific languages for administration or recognition of culturally specific images and stories.

## Second call to action: the case for digital as a clinical indicator of neurodegeneration

Long-used assessment tools continue to dominate cognitive outcomes in AD clinical trials even as digital tools offer an opportunity for lower burden, higher sensitivity, and reduced bias. Digital health tools encompass a broad swath of technologies including smartphone applications, wearable devices, computing platforms, software, and other sensors that can be applied to monitor health outcomes [49]. While the FDA has signaled strong support for digital indices that can serve as susceptibility/risk biomarkers, the pragmatic reality of current FDA approval tied to well-established clinical measures had led to a tepid response in investing in digital alternatives by both the pharmaceutical and scientific research community. In cardiovascular health, digital technologies have demonstrated utility for continuous monitoring of disease, improving patient outcomes and individual access to health data [50]. The Apple Heart Study provided evidence for the large-scale monitoring potential of digital technologies, with smartwatch-based irregular heartbeat notifications and electrocardiogram patches providing reliable home diagnoses [51]. Digital technologies can offer similar advantages in AD for large-scale monitoring [52], and may prove even more useful in evidencing the neurodegenerative process is clinically meaningful given the potential for granular capture of functional measures across domains of speech, gait, sleep, and activity that often precede clinical onset that meets diagnostic criteria [47]. While significant advances in imaging and fluid biomarkers of neurodegeneration have been developed over the past decade, despite their lack of disease specificity, identification and validation of digital clinical indicators of neurodegeneration are at an early, though promising, stage. A recent review by Polk et al. highlighted the growing body of evidence in support of the feasibility and validation of remote and unsupervised assessments for detection of subtle cognitive decline in preclinical AD. In their review, Polk et al., compared these digital assessments to more widely used cognitive assessments including the Preclinical Alzheimer’s Cognitive Composite (PACC) and other standardized neuropsychological tests. Digital cognitive assessments have shown strong correlations with plasma biomarkers, including p-tau181, GFAP, and NfL. Accuracy in classification between MCI and healthy controls, and between MCI and AD has been demonstrated cross-sectionally and longitudinally over short time periods. Thus far, discriminative accuracy for long-term longitudinal characterization of AD risk for those with and without biomarkers has not been established, but combined digital cognitive assessments and blood-based biomarkers have demonstrated promise in predicting future cognitive decline. Given that having AD pathology does not always translate to clinically expressed disease coupled with the known bias of current assessment methods and the well-documented heterogeneity in clinically meaningful outcomes (e.g., cognition and cognitively-related behaviors), our second call to action is to leverage ongoing technological advances to develop better clinical measures that evidence progression in neurodegeneration.

## Third call to action: avoid repeating history by expanding representation through scalable sensor-based devices

Our third call to action is to capitalize on continuous smartphone and wearable sensor-based assessments that can be done in a natural setting and increase inclusivity in clinical trials and other research studies [53]. Digital deployment alone will be insufficient to maximize inclusivity. Digital indices can be influenced by a multitude of sociocultural factors. Thus, it is a scientific imperative to prioritize global representation in the collection, identification, and validation of digital AD/AD and related dementias (ADRD) clinical measures. Failure to do so will perpetuate the limitations of research that has largely been conducted in high income countries, such as non-representative normative data or reliance on technologies widely inaccessible in low-resource areas. Continued proliferation of smartphones globally offers a unique opportunity to start from a globally inclusive baseline, so long as considerations of resource limitations (e.g., access to connectivity) and education/culturally/language agnostic stimuli are considered. Strategic use of technology will allow real-time monitoring of drug-treatment impact on a clinically meaningful digital “N”, which can document symptom decline with replicability possible on a globally inclusive scale.

The cost and existing uptake of digital tools compared to blood, CSF, and imaging disease markers in low and middle-income countries offers an opportunity for expanding global research and clinical integration; however, many challenges remain in avoiding the pitfalls of developing globally representative digital markers. Current barriers to inclusion of digital trial endpoints include lack of standardization in digital monitoring and best practices for the storage, management, and analysis of high volumes of digital data [54]. The lack of standard digital tools is difficult to address given the constant evolution in health technologies. In clinical practice, this lack of standardization may also present an opportunity for increased patient autonomy. Heterogeneity in the market of available digital tools presents a multitude of options ranging from active gamified cognitive testing to passive engagement monitoring which can be conducted with no burden placed on the user. Transitioning from active engagement technologies that require varied levels of staff interaction, technological fluency, and participant burden to complete set tasks, towards passive engagement technologies that capture continuous streams of data from zero-touch home-based or device-embedded sensors could yield more robust data streams with low effort longitudinal participation [52]. The promise of passive monitoring for behavioral detection is limited, however, by unresolved questions about data privacy, security, and the challenges in data storage, processing, analysis, and interpretation given the unstructured nature of continuous digital data streams. Shifting the heterogeneity in digital monitoring from a barrier to an opportunity will depend on developing robust solutions around data management and analysis, as well as patient- and physician-centered research on the accessibility, security, and privacy needs for effective clinical implementation. To fully realize this future will require efforts to advance the harmonization and analysis of large-scale digital data streams that should aim to produce device-agnostic fluidic markers of health [55]. These efforts will depend on large digital data sources with well-validated endpoints, as well as interdisciplinary cooperation to foster inclusion of open data science and machine learning analysis. Harmonization is especially challenging in an area such as digital health, where new technologies are constantly arising and aiming to innovate towards new solutions; however, achieving harmonized protocols for data collection, storage, and analysis will be crucial to avoiding the pitfalls of culturally specific cognitive assessment characterized by fractured insights on non-generalizable samples. Importantly, in this call for action, the development of pre-competitive, open source digital data management and processing tools, as well as open access data resources and results will be critical if global inclusion is to be achieved. The current dominance of proprietary hardware and software risks perpetuating the same barriers to equal opportunity science that current methods have generated. Global digital phenotyping and increased uptake of non-proprietary digital secondary outcomes, with dedicated attention to both the advanced analysis of results and qualitative feedback of users, will be needed to move digital forward towards its potential as a low-cost, accessible, early detection tool.

## Fourth call to action: reconceptualizing the A/T/N framework

Our fourth and final call to action is for a **reconceptualization of the A/T/N framework**. One that pushes to the forefront the objective of a maximally inclusive framework that is feasible worldwide, including in the lowest of research or clinical care settings. While A/T plasma biomarkers are specific to AD, complementing the current non-AD-specific “N” biomarker with a digital “N” as a clinical indicator of cognitively related behaviors and function (e.g., memory, speech, gait, balance) would attenuate the current uncertainty of whether A/T positivity will remain clinically silent. Longer-term, there is potential a digital “N” could obtain even better specificity in determining those who would most benefit from AD treatments compared to a biomarker “N”. Most studies aim to distinguish MCI or dementia from control populations without distinguishing between disease stages or subtypes. Digital measurement in its current form provides a benefit over “N” biomarkers for its potential specificity to AD. Cognitively dependent functional and behavioral measures can be digitally monitored using smartphones or other mobile internet connected devices. The opportunity to continuously evaluate these outcomes would be potentially clinically meaningful to those at high AD risk and would likely be acceptable in lieu of costly and abstract blood biomarker measurement of neurodegeneration. Increasing specificity of diagnostic tools to identify those who have or will develop clinical symptoms could significantly improve screening for clinical trials for AD. Current screen-in confirmation of AD using PET and MRI scans have added not only significant burden in study execution, but also far greater costs and greater exclusion of the at risk population, particularly in low resourced regions. Current estimated costs to bring AD preclinical treatments to FDA approval is over $5 billion [56]. Low cost, ubiquitous sensors in the form of wearable devices and smartphone applications could serve as an initial screen for clinically detectable symptoms, substantially accelerating the pace and decreasing the cost of discovering new and better AD treatments.

AD is an insidious onset disease in which the demarcation of when pathological burden translates to clinical expression is highly dependent on the assessment modality. Galvanizing the research community to consider the role of digital will push the definition of clinical expression upstream in the disease course, (e.g., to stages that are currently considered “asymptomatic”) potentially moving the focus of clinical trials that are currently centered on MCI to earlier stages. There is great excitement in the field for recently FDA approved drugs that slow the rate of cognitive decline. But how much more clinically meaningful would it be to someone at AD risk to intervene and delay or even prevent cognitive decline? Research has repeatedly indicated that delaying onset of symptoms by 5 years can reduce overall risk for disease by 50 % [57]. We argue that digital technologies offer an opportunity to re-evaluate current definitions of clinical meaningfulness and consider what new directions of intervention will emerge when at-risk patients and their physicians can consider prevention of disease as an alternative to treatment.

Further investment in and evaluation of digital markers could lead to highly accurate prognostic indications of AD and its etiological subtypes. Increased sensitivity and specificity of change far earlier in the AD disease pathway are ambitious but possible objectives. To meet this bold vision will require a transition from the practice of collecting single modality digital phenotypes that are often analyzed as relatively static derived measures towards collection of rich, multimodal digital data streams [58], that are analyzed in their native dynamic and fluid format. Rigorous and transparent testing for reproducibility and replicability will be needed, with prioritization of using foundational open-source tools. An early focus on pre-competitive approaches will be important because the considerable challenge of research keeping pace with the rapid and continuous evolution of digital technology and artificial intelligence (AI). These constant advances in digital measurement potential, and corresponding analytic capacity will lead to compressed timelines for monitoring disease indicators, providing clinical practice with a real time prognostic marker that has stronger sensitivity and specificity of clinically meaningful change. “N” markers today still provide value in the confirmation of conversion risk through structural change, although the cost of additional testing may not be justifiable if the potential for digital monitoring is fully realized.

Everything people do is through their brains, and cognitively related skills are continuously expressed through behaviors such as speech, gesture, and movement. It is time to harness the power of digital to capture these clinically meaningful measures that are critical to determining efficacy of any AD treatment. But to do so will require the same scientific creativity that has led to breakthroughs in AD imaging, CSF and plasma biomarkers of A/T and apply them to the development and validation of the digital “N”.

## Funding

This work was informed by research supported by the American Heart Association (20SFRN35360180), the National Institute on Aging (2P30AG013846, AG062109, AG068753, AG072654, the Alzheimer’s Drug Discovery Foundation (201902-2017835), the TMCity Foundation, and the Davos Alzheimer’s Collaborative.

## Consent statement

Consent was not necessary for the purposes of this perspective piece.

## CRediT authorship contribution statement

**Rhoda Au:** Writing – review & editing, Writing – original draft, Supervision, Conceptualization. **Zachary Popp:** Writing – review & editing, Writing – original draft. **Spencer Low:** Writing – review & editing, Writing – original draft. **Nicholas J. Ashton:** Writing – review & editing, Writing – original draft. **Henrik Zetterberg:** Writing – review & editing, Writing – original draft.

## Declaration of interests

The authors declare the following financial interests/personal relationships which may be considered as potential competing interests: Rhoda Au reports a relationship with Novo Nordisk Inc that includes: consulting or advisory, speaking and lecture fees, and travel reimbursement. Rhoda Au reports a relationship with Signant Health that includes: consulting or advisory. If there are other authors, they declare that they have no known competing financial interests or personal relationships that could have appeared to influence the work reported in this paper.
